# Acting between guidelines and reality- an interview study exploring the strategies of first line managers in patient safety work

**DOI:** 10.1186/s12913-020-06042-3

**Published:** 2021-01-08

**Authors:** Mats Hedsköld, Magna Andreen Sachs, Torleif Rosander, Mia von Knorring, Karin Pukk Härenstam

**Affiliations:** 1 Medical Management Centre, Department of Learning, Informatics, Management and Ethics (LIME), Karolinska Institutet, 171 77 Stockholm, Sweden; 2 Department of Anaesthesiology and intensive care, Södersjukhuset, Stockholm Region Sweden; 3 Department of Paediatric Emergency Care, Astrid Lindgren’s Children’s’ Hospital, Karolinska University Hospital, Stockholm, Stockholm Region Sweden

**Keywords:** First line managers, Patient safety, Patient safety culture, Intensive care, Hospital survey on patient safety culture

## Abstract

**Background:**

Safety culture can be described and understood through its manifestations in the organization as artefacts, espoused values and basic underlying assumptions and is strongly related to leadership-yet it remains elusive as a concept. Even if the literature points to leadership as an important factor for creating and sustaining a mature safety culture, little is known about how the safety work of first line managers’ is done and how they balance the different and often conflicting organizational goals in everyday practice. The purpose of this study was to explore how health care first line managers perceive their role and how they promote patient safety and patient safety culture in their units.

**Methods:**

Interview study with first line managers in intensive care units in eight different hospitals located in the middle of Sweden. An inductive qualitative content analysis approach was used, this was then followed by a deductive analysis of the strategies informed by constructs from High reliability organizations.

**Results:**

We present how first line managers view their role in patient safety and exemplify concrete strategies by which managers promote patient safety in everyday work.

**Conclusions:**

Our study shows the central role of front-line managers in organizing for safe care and creating a culture for patient safety. Although promoted widely in Swedish healthcare at the time for the interviews, the HSOPSC was not mentioned by the managers as a central source of information on the unit’s safety culture.

**Supplementary Information:**

The online version contains supplementary material available at 10.1186/s12913-020-06042-3.

## Background

Health care is a complex socio-technological system where the individual and collective work practices not only strive to produce the desired outcomes of the system, but also where system safety emerges [1]. The concept of safety culture was coined in the aftermath of the Chernobyl accident and the Challenger crash in 1986 and has been defined for healthcare as “The shared values, attitudes and behavioral norms that determine the degree to which all organizational members direct their attention and actions toward minimizing patient harm during delivery of care” [2]. Safety culture can be described and understood through its manifestations in the organisation as artefacts, espoused values and basic underlying assumptions [3] and is strongly related to leadership [4, 5] yet it remains elusive as a concept.

In Sweden, based on the Swedish patient safety law [6] the health care providers (whether county-owned or contracted private) are responsible for patient safety and should conduct systematic patient safety work. Tasks aiming at executing this imposed regulation are delegated to first line managers, who also are responsible for staffing of the unit round the clock and ensuring that work is done according to rules and that the mission and goals of the organization are adhered to. The work with improving patient safety and patient safety culture was intensified when a patient safety law [6] was launched along with a government-supported financial incentive plan for 2011–2014 aiming at improved patient safety. One of the performance-based requirements of this incentive plan was to measure patient safety culture and – based on the outcome of the measurements – make action plans and initiate improvement projects aiming at development of the patient safety culture. A Swedish version of one of the most spread tools for the purpose of measuring patient safety culture - Hospital Survey on Patient Safety Culture (HSOPSC) developed by the Agency for Healthcare Research and Quality - had earlier been translated and reworked to Swedish conditions [7] and was used during these years for repeated measurements in hospitals and health care organizations in Sweden. Although widely implemented in Swedish healthcare, the actual use of the survey in local patient safety initiatives has not been explored.

The often-conflicting organizational goals under which healthcare happens put pressures on first line managers and staff [8]. Under these conditions, as highlighted by the challenger crash, shared values and work practices can drift towards acceptance of unsafe behaviors and violations of safety protocols. This phenomenon has been called “normalization of deviance” [9].

The important and ambidextrous role of middle and front line managers in implementing patient safety culture is argued to be central [10], but in contrast to the well-developed theoretical models, there is still a gap in the systems safety field in explaining how to move from theory to everyday management [11]. High reliability Theory (HRT), developed from the study of organizations that achieve quality and safety goals in spite of hazardous processes, has been suggested as a frame to explore and understand safety practices in Health Care [12]. HRT emphasises leadership and culture and points to a number of aspects that are important for safety in high reliability organisations (HROs) - deference to expertise, resilience, sensitivity to operations, preoccupation with failure and shared baselines [13]. HRT approaches have been suggested as a complimentary approach to other safety theories in understanding how health care organizations could manage the tensions between organizational stability and change [14].

In summary, even if the literature points to leadership as an important factor for creating and sustaining a mature safety culture, and theoretical constructs for important aspects exist, little is known about how first line managers’ operationalize these in everyday practice [15, 16].

### Aim

To explore how health care first line managers perceive their role and how they promote patient safety and patient safety culture in their units.

## Methods

### Participants and settings

Eight first line managers working in intensive care units (ICU) in eight different hospitals located in the middle of Sweden were interviewed.

Our selection of participating hospitals and ICU units within these hospitals was based on a purposive sample where we aimed for maximum variation in the sample [17]. The hospitals differed in size from smaller district hospitals to larger county and region hospitals, of which some were university hospitals providing highly specialized care. The ICU ´s varied in size and were either focusing on acute coronary care, postoperative or trauma care. The hospitals and the ICU first line managers of this study were thus selected to get a purposive sample based on variation in hospital size and type of ICU and organizational culture in which the managers worked.

ICU first line managers were chosen for the study since intensive care is known to be an example of medical care with high risk of harm being done to the patients due to the advanced technology used in ICU:s and the frailness of the patients [18]. The hospitals differed in size from smaller district hospitals to larger county and region hospitals, of which some were university hospitals providing highly specialized care. The ICU ´s varied in size and were either focusing on acute coronary care, postoperative or trauma care. The ICU first line managers of this study were thus selected to get a purposive sample based on variation in size and type of ICU. An invitation to participate in the study was sent by email to the managers at 8 ICU units that met these selection criteria.

Inclusion criteria for interviewees were: 1) two years or more of experience as first-line manager and 2) the unit where they held their position should have participated in a patient safety culture survey between 2009 and 2011 using the HSOPSC instrument. In case the unit approached held more than one first line manager, the invitation to participate in the study was addressed to the manager whose name appeared first on the electronic contact list. In the mail the background and purpose of the study was described, and a presentation of the research team was given. Also, a letter was enclosed stating that participating in the interview was voluntary and that information given during the interview was to be regarded as confidential material. Also, the letter contained information about how data would be used and stored. All persons who received this invitation letter agreed to participate in the study.

Among the participants, the experience as a first-line manager, ranged from two to ten years (average 4.6, median 4.5). Six of the eight participants were nurses and two were physicians. Further, six of the interviewees were women. The number of staffs per unit ranged between 19 and 140 (average 91, median 115).

## Data collection

A semi-structured interview guide with open-ended questions was constructed based on current knowledge and discussions among the researchers (Additional file 1). The guide was revised after the two first interviews to better correspond to the aim. All interviews are included in the analysis. The interviews were conducted in 2013.

The interviews took place at the informant’s own workplace or close to this place except one interview which was held over the telephone for practical reasons. All interviews were conducted under written informed consent, lasted between 30 and 55 min, were digitally recorded, and transcribed verbatim by the interviewer shortly after each interview.

## Data analysis

The research group consist of both managers, practitioners and researchers and this has allowed for reflexivity and iterative discussions of the themes and results.

An abductive qualitative content analysis approach, inspired by Graneheim and Lundman [19, 20] was used to analyze data. The analysis used an initial inductive stage where categories were identified. In this first step, all interviews were read by all authors to obtain an in-depth understanding of the data. The authors then collectively identified meaning units in one of the interviews and discussed ideas about categories and themes. In the next step, two of the authors (MH and MAS) reread all transcripts and marked all passages – meaning units – that were related to the aim and research questions. These meaning units were then condensed and coded close to the manifest content and through a process of negotiated consensus within the research group classified into categories and subcategories. The codes and their relations were frequently discussed in the research team and sorted into tentative sub- and main categories. Last, a deductive comparison of relations between the categories describing strategies used by the managers to theoretical constructs drawn from research on high reliability organizations was made to provide new insights to the findings (Table 1) [20].

**Table 1:** Example of the analysis in three-steps: the inductive content analysis going from *meaning unit, category and then the deductive interpretation of the category based on HRO construct*

Meaning unit	Subcategory	Deductive interpretation
” Two weeks out of four I am dressed in hospital working clothes and I take part in daily work. The other two weeks I wear private clothes and work administratively. When dressed in working clothes, staff members always come up to me and give comments and reflections on experiences and observations during work / … / and they generally express a wish that I would be present like that every day.” (IP7)	Being present in daily work and listening to staff	The managers give many examples of how they operationalise being ***sensitive to operations*** by being present in the daily work.

The developed analytical themes (by MH, MAS and KPH) were regularly discussed in the larger research team to test them empirically and thus enhance credibility. To test the relevance over time, since the interviews are conducted in 2013, the themes were presented to the members of the research group that work in the ICU setting or with safety management (KPH, TR) iteratively over time.

In the Results section below, quotations from the interviews are used to illustrate the findings. A number is given within parenthesis that connects the quotation to a specific interviewee. In the quotes / … / indicates omissions and [] additions of text. Changes have only been made to enhance readability and have not altered the content of the quotations. All quotes have been translated from Swedish.

## Results

First, we describe how managers saw their role in developing and sustaining a culture for patient safety and the role of patient safety culture surveys in guiding patient safety work. Second, we present the results from the deductive analysis that yielded five themes describing strategies that managers used to promote patient safety and patient safety culture within their units: Valuing and developing healthcare professionals´ expertise, Organizing for resilience, Being present and setting a good example in daily work, Encouraging individual and organizational learning from incidence reporting and Balancing adherence to and questioning of standardized operative procedures.

### A. Developing and sustaining a culture for patient safety

In general, the first line managers were convinced that they personally had a great impact on patient safety and patient safety culture within the unit as was phrased by one of them in the following way:

*" I believe I play a tremendous role in promoting patient safety”* (IP2).

The managers also expressed a deep commitment to patient safety issues and viewed these missions as their main duties as exemplified by this quotation:

*" Looking at my job in a wider perspective I think it is all about strengthening patient safety”* (IP5).

The managers strongly believed they played an important role in the development of a positive patient safety culture which also was looked upon as a never-ending mission. The importance of their personal attitudes in situations where they, themselves, might have put patients at risk or even worse, made them experience harm, was described as essential for building a supportive safety culture. One of the managers formulated this as follows:

*" Yes, I do think it is very important that you dare to speak up about your own mistakes. Because I think that’s what it’s all about. To have psychological safety – yes, dare to show your own mistakes.”* (IP6).

Their way of fulfilling this mission was described in terms of keeping up correct routines and constantly encouraging incidence reporting. Speaking up around adverse events was said to promote safety by raising the level of attention to risks in daily work and underlining the necessity of adherence to safe work practices. The informants also stated that they played an important role in encouraging and confirming good patient safety practice and especially in not condemning errors and mistakes- as expressed by one of the managers:

*" What is also important is to set a good example …*. *create a climate where everybody is comfortable with speaking up … for instance an assistant nurse might say to a senior doctor that that is not the correct way of doing this”* (IP8).

Another manager reflected on the need of humbleness and supporting development of a blame-free environment by role modelling how staff could approach a situation where they felt uncertain about the correct way of doing things, in the following way:

*" I do not hesitate to say” oh, I do not actually know how to do this. I have to check it up first!” or” this was not so well done / … /I should have known better”. I am very prone to use these words / … / in order to show humbleness.”* IP6.

The managers stressed the importance of emphasizing patient safety as everybody’s responsibility and recognizing the patient safety aspects in every detail of the job. As one of them phrased it:

*" Everything we do, all our activities, should be done with patient safety in focus.”* (IP7).

The managers saw promoting a positive and supportive working climate as an important task:

*" If they do not feel comfortable and like it here then there is no joy in going to work and that effects how patients are treated”* (IP3).

Finally, having routines and a structure in place for supporting staff members after they have been involved in an adverse event was stressed as an important factor in building a good working climate. One of the managers described this in the following way:

*" We care for the staff member who has been involved in an adverse event / … / sit down and talk / … / listen / … / provide support/ … /”* (IP1).

### B. Experience of using HSOPSC surveys to guide patient safety improvement work

All the units had participated in the HSOPSC survey and half of the units had already completed it a second time. None of the managers described any systematic use of the patient safety culture measurements as a tool or a guide in their work to promote and improve patient safety culture and patient safety. One of the reasons expressed for not using the survey were that they did not know how to interpret the results.

*“Honestly, I actually do not really remember but let me think /../.*

*There was some feedback, we talked about it and the hospital leadership still referred to it occasionally. I think that results were posted on the intranet, well that was it. I remember that when we gave feedback to the staff, I and looked at the bars and circles online. Yes, and as I said, the results were online”/../. (IP2).*

The managers admitted that their motivation for working with HSOPSC was low, despite their focus on constantly improving patient safety and awareness of the importance of a strong patient safety culture. Also, the managers described difficulties in getting feedback on survey outcomes and said that they were not given the tools and training in how to interpret and act upon the results. This situation was described in this way by one of the managers:

*” I would have liked to have a constructive discussion about all the questions …. also, a plan … what are we aiming at? What are our goals? And a toolbox … how should I interpret this picture... the spider diagram … what am I supposed to do with it?* (IP7).

Measurements were said simply to be done to receive financial revenue through the national initiative plan, not as a part of a goal-oriented local improvement plan.

The managers expressed that they were not involved in decisions on when to conduct a survey or in the communication of survey results. Timing of patient safety culture surveys seemed to be set by management higher up in the health care organization without any consultation with first line managers to capture when and what information was needed in order to support and further develop ongoing patient safety initiatives.

Other explanations raised by the managers was the lack of support from higher management in how to improve safety culture. The only exception was one manager who described that the Chief Medical Officer at the hospital had demanded that the survey outcome should be used as a basis for local patient safety improvement plans. Thus, action plans for improved adherence to handoff routines and improved feedback on incidence reporting were developed as an effect of poor outcomes in these areas in the recent patient safety culture survey.

*“/ … /all units were forced to come up with an action plan based on the survey outcome / … /to show that you had done something.”* (IP3).

### C. Strategies used by managers to promote patient safety and patient safety culture

#### C1. Valuing and developing health care professional’s expertise

The managers emphasized the importance of expertise and actively supported the development of health care professionals´ competencies. Many managers saw noticing and recognizing the competencies of individual staff as central task linked to the resilience of the ICU.

*“Also, make use of all competencies that each one among staff members possess / … /which makes them feel acknowledged for who they are / … / what their contributions mean to the whole picture / … /”* (IP5).

The managers emphasized the importance of supporting continuous development of staff members, both in medical care and in-patient safety issues, for them to deliver safe care. The managers gave examples of how they incorporated in daily respectful conversations between them as leaders and the unit staff by providing constructive suggestions when/where appropriate and inviting reciprocal recommendations for professional growth. Using the expertise of staff members was also seen as an important strategy for improvement. Sometimes experts were invited to present new evidence or information calling for change of routines. This was said to have a greater impact than the manager giving the information.

*” For example, if staff adherence to hygiene routines has been studied in our unit it is much better if an outside expert, involved in the study, comes and gives feedback on the results and not me or anyone else within the unit. Listening and learning will be much better.”* (IP1).

#### C2. Organizing for resilience

The everyday organizing of clinical work seems to dominate the work of the managers in this study. One central aspect in promoting a patient safety, was to secure that enough staff were present during each shift and ensuring that necessary competences were represented by the team.

*” It is in the end a patient safety issue that staff is well trained and competent”* (IP4).

The managers’ argued that focusing on scheduling the right competencies for a shift was an important precondition for the team’s capability to manage everyday work as well as to reorganize and respond to a variety of expected and unexpected situations in the complex ICU environment without deviating from safe practice. As one manager stated:

*” I think that a too heavy workload is absolutely devastating [for patient safety] because then you start to prioritize [and ignore basic safety routines]. And when you do that you are dissatisfied with your work”* (IP7).

#### C3. Being present and setting a good example in daily work

The managers stated unanimously that it was of great importance to be present and, as much as possible, take part in daily, clinical work because, by so doing, opportunities for picking up problems which might not otherwise have been communicated to the manager were created. One of the managers said:

*” Two weeks out of four I am dressed in hospital working clothes and I take part in daily work. The other two weeks I wear private clothes and work administratively. When dressed in working clothes, staff members always come up to me and give comments and reflections on experiences and observations during work / … / and they generally express a wish that I would be present like that every day.”* (IP7).

Also, when the managers participated in daily work potential risks were spotted by the manager him- or herself as e.g. shifts in routines having occurred and current opinions on how to execute certain care procedures having occurred:

*” It is quite another thing …*. I *sit in the coffee room as one of the staff and listen and then they can ask, and I sort of get a deeper understanding and see it from their perspective …*” (IP6).

The saw themselves as role models and that their way of acting and reacting in routine work as well as critical situations was copied by their staff.

*” Always setting a good example / … / in clinical work, in patient care/ … / For instance never deter from basic hygiene rules / … / but also in my attitudes / … / expressing a will to communicate and to speak up / … / demonstrating an attitude / … /”* (IP3).

Also, it gave the managers excellent opportunities to exert a direct influence on attitudes and manners, as was expressed by one of the managers in this way:

*” Communication / … / and daring to take part in daily work, showing how things should be done / … / that is really the most important part* [*of patient safety work*]*”* (IP8).

#### C4. Encouraging individual and organizational learning from incidence reporting

Fostering an active incidence reporting, preferably via the electronic system was mentioned as an important part of the first line managers´ efforts to maintain and develop patient safety within the unit. One of the informants described the necessity of sharing information on adverse events like this:

” *It is done to make staff aware about what has happened, and that this is something that we need to be extra careful about”* (IP1).

Analysis of the reports was generally handled by one or more members of the unit including – in case of a serious incident – the head of the department. The suggestions for improvement emerging from these analyses were subsequently implemented through existing forums. The first line managers expressed an ambition to provide both a written feedback to each individual employee who had filed a report and also, orally, inform all staff members, in conjunction with staff meetings, about the outcome of the analysis of the incidence report and what measures that had been taken or were to be taken in order to prevent this incidence and similar others from occurring again. Due to an overload of reports, however, the ambition to analyze and give feedback on all reports that had been filed was not always possible to live up to. Those reports which were regarded as “minor” incidents thus had to be left unattended. This was generally regarded as a failure since windows for improvement might have been left unused.

Further, to deepen the understanding of how patient safety is treated, the employees were not only provided with feedback from analyses of adverse events but also invited to take part in root cause analyses (RCA) teams or improvement efforts in order to learn the methodology and get a deeper understanding of the risks in health care and how to prevent harm.

*” I think a lot of how I can reach as many as possible of my staff about ongoing patient safety work, but it is not that easy. So, what I can do is to tell them about the outcomes of the root cause analyses that we have done / … /. Also, I try to get as many members of my staff as possible included in root cause analyses teams as a learning opportunity”* (IP6).

Sometimes, groups of employees were given the opportunity to analyze an incident on their own and afterwards compare their conclusions and suggestions with those of the formal RCA group. Involving co-workers in RCA activities or in group discussions around the results of such analyses was said to stimulate creativity among staff members i.e. further develop their capacity for solving problems and finding ways to provide safer care. This was described in the following way by one of the first line managers:

“*It is formally me who makes the final registration in the system, but in this way we get more staff members engaged in the discussion on how to find good solutions on how to move ahead”* (IP7).

The managers, however, regretted that their aim to create continuous learning often rested unattended since little or no time was set aside for performing follow up on changes that had been made. One of the managers formulated it like this:

*” We are good at starting new projects, but we never evaluate the ones that we already have started”* IP5.

#### C5. Balancing adherence to and questioning of standardized operative procedures

The first line managers described their responsibility for ensuring that constantly updated documents on evidence based, standard operative procedures were available and adhered to in daily work. One manager emphasized the role of these guidelines in providing high quality care to patients in the following way:

*” Updating guidelines is also a part of the improvement work. Patients should be treated in accordance with new evidence and not according to old rules that have never been revised”* (IP5).

Another manager expressed great personal pride in ensuring that guidelines were updated in the following way:

*” For me it is important that we have updated guidelines and memos to follow. I am very proud that we have a good structure around that”* (IP4).

However, the mangers described how they found the task to keep all guidelines updated, communicated and adhered to challenging. They described that they sometimes took the responsibility not to introduce documents on standard operative procedures sent out from top management of the hospital because the procedures advocated from this level according to their opinion were not always applicable to intensive care. One manager said:

*” It is like this: the kind of medical care that we provide does not always fit with the instructions sent out from the top.”* (IP1).

Also, in certain situations, when unexpected things happened and no written guidelines fully applied to the situation, the managers expressed a wish for employees not to be too dependent on guidelines but instead use their own creativity and try to focus on the best way of solving the situation without harm being done and then, afterwards, decide whether a new guideline was needed or the old one was obsolete and in need of updating.

*” We encourage staff members to act like this and then afterwards to engage in discussions and analysis of the situation. Maybe guidelines applicable to this type of situation need to be changed or there is need for a new guideline?”* (IP2).

On the other hand, too creative staff members could also pose a risk and the managers underlined the importance of matching creativity with an explicit process for how to eventually decide on and launch a new routine in order to prevent uncontrolled methods and ways of working entering the scene. One of the managers formulated it like this:

*” This [strategy] leads to my staff being fairly good at solving problems. But this can of course be hazardous, so you must have structures for how to handle such situations”* (IP2).

## Discussion

The inductive analysis yielded two themes that gave insights into the managers thoughts on their role in developing and sustaining a culture for patient safety, and their experiences of using the safety culture assessments. To further illuminate the findings a deductive analysis was performed using constructs from HRO’s that yielded five themes describing strategies that managers used to promote patient safety and patient safety culture within their units: Valuing and developing healthcare professionals expertise, Organizing for resilience, Being present and setting a good example in daily work, Encouraging individual and organizational learning from incidence reporting and Balancing adherence to and questioning of standardized operative procedures.

The managers in our study shared their insight, desire and commitment to work with patient safety and patient safety culture and gave examples of how they strive to embody the different aspects of safety culture as defined in the introduction of this study [2]. Their insights highlighted how safety culture for them was not a construct but something that is actively being constructed in all the different meetings and exchanges that the first line managers have during a workday. A study from the Veteran Administration hospitals report similar findings; they highlight leadership of managers at daily briefings before the start of shifts or procedures in clinical areas as a mechanism of establishing and maintaining safety practices [21]. The managers also expressed the importance of promoting a safe environment where staff members feel seen and listened to. This focus on supporting the staff in their everyday work to create a good work environment seems to be central. This intuitive connection expressed by the managers in our study is mirrored by a recently published systematic review by Braithwaite et al. who found that positive associations exist between workplace culture and patient outcomes [22]. This link between employee safety and systems safety has been described in other industries [23] and is highlighted as an important precondition for safety culture and learning [24].

In our study, to our initial surprise, none of the managers used the results of the safety culture survey to guide their work. The reasons expressed for not using the survey were that they did not know how to interpret and improve the results. Other explanations raised by the managers was the lack of support from higher management in how to make use of the results in order to improve safety culture. The importance of the capacity of managers to operationalise and tailor national safety and quality initiatives to their context has been corroborated by other studies [25].

Recent studies highlight the challenge of middle and first line managers to make sense of the safety culture surveys and how new constructs are being developed that are closer to the reality of safety management as described by the managers in our study [13]. One such construct used in this study is that of high reliability organisation (HRO)- deference to expertise, resilience, sensitivity to operations, preoccupation with failure and shared baselines [13]. The emerging themes from the deductive analysis show examples of how managers operationalised several of the aspects of HROs in their everyday leadership.

They emphasise the importance of *deference to expertise* by *valuing and developing health care professionals´ expertise*. They do this by supporting the development of competency and by recognizing work well done. They also describe how constructive suggestions when/where appropriate, and reciprocal recommendations for professional growth, can be incorporated in daily respectful conversations between them as leaders and the unit staff. Studies have highlighted that providing more feedback about performance may be a welcome process for the staff in clinical areas [26].

The everyday *organising* of clinical work *for resilience* seems to dominate the work of the managers in this study. Many managers saw noticing, recognising and developing the competencies of individual staff as central task linked to the resilience of the ICU. The managers’ focus on knowing the staffs capabilities and scheduling the right competencies can be seen as an important precondition for the team’s capability to manage everyday work as well as to reorganise and respond to a variety of expected and unexpected situations in the complex ICU environment. Making such early investments in the competencies of front-line staff have been highlighted as important for creating capacity for staff to be able to notice risks and as a precondition for organisational resilience [27].

The managers give many examples of how they operationalise being *sensitive to operations* by *being present and setting a good example in the daily work*. The importance of being present and visible as a leader in the sharp end while working with improvement of safety has been highlighted earlier [5]. Savage et al. showed how the managers played a central role in the redesign of work practices by actively participating in and enabling staff to participate in the improvement teams, championing Crew Resource Management (CRM) and participating in the redesign of work practices. This presence also led to improvements in technical and non-technical skills as well as to safety culture but also that the staff perceived the leader’s commitment to safety more clearly [28].

The interviewees in our study gave examples of *preoccupation with failure* in how they described their efforts for *encouraging individual and organizational learning from incidence reporting.* The strategies they shared were similar to those put forward by Gandhi et al. 2017 [29], i.e. counteracting any form of punishment of human errors but at the same time holding staff members accountable for their actions or failures to act. The shift in perspective from individual learning from failure to strategic or organisational learning seemed to be perhaps challenged by the immediate task to get the unit to function; several of the managers expressed how they struggled with closing the loop with regard to learning from and reorganising work based on adverse events.

Adherence to shared baselines has recently been suggested as an important trait in HROs [13]. Several managers expressed pride in having updated guidelines and standardised operative procedures and in working with staff so that they feel informed when changes are introduced. However, the focus on adherence to guidelines and standardised operative procedures was balanced by the comments that emphasised the importance of questioning the feasibility of guidelines in the complex ICU context. An important point made within the resilience engineering perspective is that safety can be managed by structure and control on one hand (i.e. Safety − 1) and adaptive behaviour on the other (Safety − 2) [30].

The managers gave examples of how teams adapt their work to changing preconditions or to unexpected developments and that they perceive their role as acting between promoting structure on the one hand and encouraging innovation and learning on the other hand. The awareness of this balancing and the need to stop any drift in practice that could pose a risk to patient safety seemed to be a central task [10] and this constant mindful balancing act seems to be a central strategy used by the managers in this study in preventing normalisation of deviance [9]. The work practices of the units were not only challenged by the adaptations made by staff. The managers describe how reorganisations of work or new routines promoted by higher management can be disruptive if they are misaligned with the ICU context. A recent Norwegian study did indeed show how reorganisation of ICU units can affect safety culture negatively [31]. Shielding their units from unnecessary change or unaligned guidelines is mentioned as an important part of the job by the managers. This ambidextrous role in change and innovation activities of middle managers have been proposed to play an important role– promoting both exploration and exploitation of new guidelines and innovations to clinical practice and guidelines [10, 25].

## Limitations

There are several limitations to this study that affects the applicability to practice of our findings. Firstly, the interviewees all worked in ICUs as managers thus all representing the same perspective on the subject thus affecting the transferability of our findings to other contexts. Secondly, only eight managers were interviewed. The managers were chosen to presumably represent eight different safety cultures. Our selection of participating hospitals and ICU units within these hospitals was based on a purposive sample where we aimed for maximum variation in the sample. There was variability about the size of the ICUs as well as the experience of the managers and when analysing the data we had as well as a richness and variability to the answers with the current sample size. Also, a basic assumption laying behind the selection of participants in the study was that safety culture is dependent on the organization in which it is embedded rather than on an individual. The interviewees where foremost representatives of their contexts. Thirdly, the study was conducted in 2013 and although there has been no significant changes done to the organization of intensive care or the role of first line managers in Sweden nor any new local or national initiatives addressing safety culture since this study was conducted the pre-understanding of safety among managers may have evolved since then. Trustworthiness during data analysis was addressed by regular peer-check and in seminars with the wider research group. The findings have been iteratively discussed with the members of the group that have managerial roles to test for credibility and so that they do not reflect the interpretations of just one researcher. Transferability was addressed by leaving an audit trail of extracts from the data in the report so that readers from other fields can evaluate if the results are transferable to their respective contexts. Table 1 of the methodological section provides a trail of how interpretations of data were made.

## Conclusions

Our study shows the central role of front-line managers in designing the everyday work in the ICU and provides examples of how safety work is done in ICUs thus contributing to closing the gap between safety theory and practice. The safety work of first line managers is central and the preconditions for it as well as building knowledge of how to support it needs further exploration.

## Supplementary Information

**Additional file 1.**

## Data Availability

The datasets used and/or analysed during the current study are available from the corresponding author on reasonable request.

## Abbreviations

ICU: Intensive Care Unit
IP: Interview Person
HRO: High reliability Organisations
HSOPSC: Hospital Survey on Patient Safety Culture
RCA: Root Cause Analysis
CRM: Crew Resource Management
