# Supplementary material for: Acting between guidelines and reality- an interview study exploring the strategies of first line managers in patient safety work
Source: BMC Health Serv Res. 2021 Jan 8;21:48. doi: 10.1186/s12913-020-06042-3 (PMC7796601; doi:10.1186/s12913-020-06042-3)
Supplement: Supplementary file 1 — Additional file 1. [file 12913_2020_6042_MOESM1_ESM.docx]

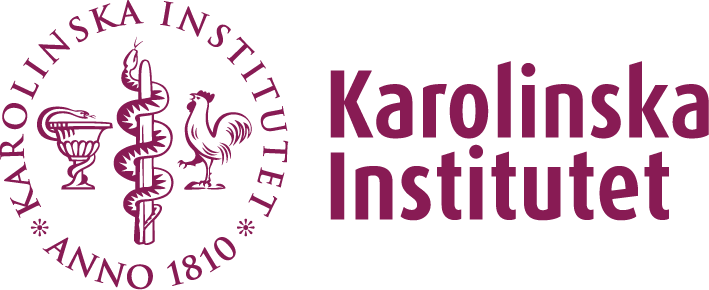


Department of Learning, Informatics, Management and Ethics ,

Medical Management Centrum (MMC)

**Information about the study**

The purpose of this interview is to examine first-line managers' experiences of their own significance and impact on patient safety work, as well as to examine how to explore and how the unit has worked with the results from the measurements of the patient safety culture.

**Informed consent**

**Background questions**

Age?

Gender?

How many years of experience do you have as managers of this unit?

What is your clinical background?

How many employees do you have at the unit? Which professions?

How many of the staff are you managing?

**First, I would like to learn more about the patient safety culture survey?**

When/How often has your unit participated in patient safety culture measurements?

Have the results been reported back to the managers and staff? How?

Who presented the results from the measurements?

Did you get any material from the hospital management to guide the presenatations?

How were the results presented to the unit managers?

Do you recall the results or any areas of improvement suggesterd by the survey?

Have any of the results been intergrated in the patient safety strategies for the unit? How?

**How do you report adverse events?**

Describe the adverse event reporting system?

Who manages the reports?

**If we now focus more on your role- Could you describe how you perceive your role as a manager in working with patient safety?**

Do you see differences in how different categories of staff perceive your role?

**Could you give any examples of how you affect the patient safety work?**

**How do you make your role as unit manager visible in working with patient safety?**

**Describe what you mean by safety culture and what role you have as a manager in influencing safety culture**

**What aspects would you raise when thinking about how managers affect safety culture in healthcare?**

**What strategies do you use in your role as a manger?**

**What strategies do you have when approaching staff that are reluctant to follow guidelines or act in a manner that influences the safety culture in a negative way?**

**Are there any other aspects that we have not touched upon during the interview that you would like to raise?**

Thank you!
